# Structural and Functional Equivalency Between Lyopreserved and Cryopreserved Chorions with Viable Cells

**DOI:** 10.1089/wound.2019.1041

**Published:** 2020-09-17

**Authors:** Vimal Jacob, Nicholas Johnson, Anne Lerch, Brielle Jones, Sandeep Dhall, Malathi Sathyamoorthy, Alla Danilkovitch

**Affiliations:** Research and Development, Osiris Therapeutics, Inc., Columbia, Maryland.

**Keywords:** viable lyopreserved chorion, viable cryopreserved chorion, tissue preservation, lyopreservation, cryopreservation, fibrosis

## Abstract

**Objective:** Clinical studies have demonstrated that the use of cryopreserved amnion or trophoblast (TR)-free chorion, containing viable cells, in the treatment of chronic wounds results in high rate of wound closure. Recently, a new lyopreservation method has been developed for preservation of amnion that also retains the endogenous viable cells. The objective of this study was to use this method for lyopreservation of TR-free chorionic membrane (viable lyopreserved chorionic membrane [VLCM]) and compare it with the viable cryopreserved chorionic membrane (VCCM). A second objective was to investigate the immunogenicity of chorion, an important question that has not been fully addressed.

**Approach:** Chorion immunogenicity was tested *in vitro* in a mixed lymphocyte reaction and lipopolysaccharide (LPS) challenge assay, and *in vivo* in a mouse subcutaneous pocket implantation model. VLCM tissue structure was assessed histologically, growth factor content by multiplex assay, and cell viability by LIVE/DEAD cell fluorescent staining. Inhibition of tumor necrosis factor α secretion by LPS-activated THP-1 cells and endothelial cell tubule formation assays were performed to evaluate the anti-inflammatory and proangiogenic properties, respectively. An *in vivo* rabbit abdominal adhesion model was used to evaluate the antifibrotic properties.

**Results:** Chorionic membrane without trophoblast (CM) was shown to be nonimmunogenic. Tissue architecture, growth factors, and cell viability of fresh CM were maintained in VLCM and VCCM. *In vitro* studies showed that anti-inflammatory and angiogenic properties were retained in VLCM. Furthermore, VLCM prevents formation of postsurgical adhesions in a rabbit abdominal surgical adhesion model.

**Innovation:** Characterization of structural and functional properties of VLCM is reported for the first time.

**Conclusion:** Similar to VCCM, VLCM retains native components of fresh CM, including collagen-rich extracellular matrix, growth factors, and viable cells. *In vitro* and *in vivo* models demonstrate that VLCM is anti-inflammatory, proangiogenic and antifibrotic. Results of this study support the structural and functional equivalency between VLCM and VCCM.

**Figure:**
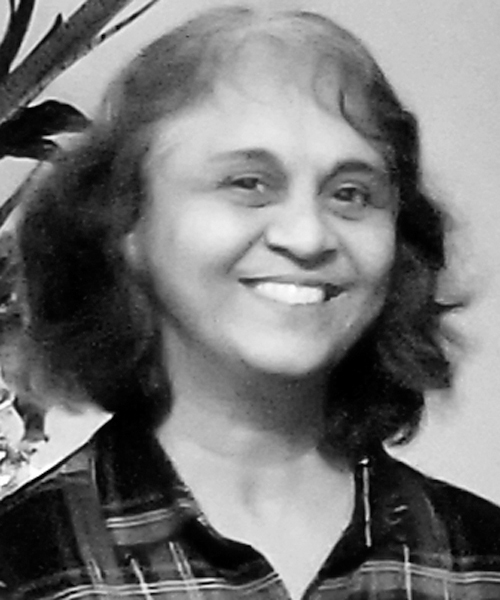
**Malathi Sathyamoorthy, PhD**

**Figure:**
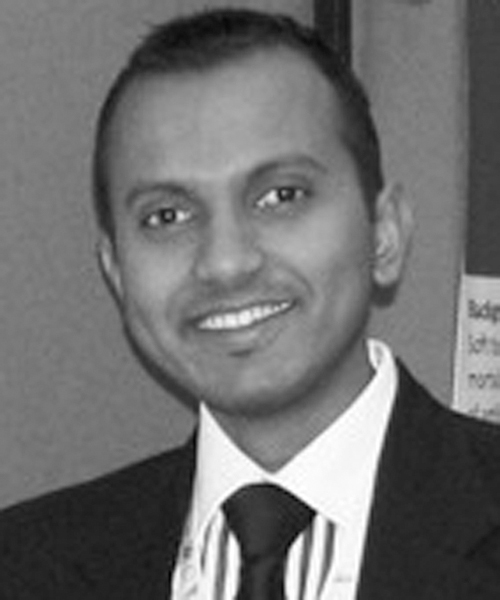
**Sandeep Dhall, PhD**

## Introduction

The human placental membranes have anti-inflammatory, antimicrobial, proangiogenic, and antifibrotic properties, making both ideal natural biomaterials to cover wounds or surgical sites.^1–3^ These properties are attributed to the placental membrane components: a collagen-rich extracellular matrix, a native cocktail of cytokines and growth factors, and endogenous viable cells, including mesenchymal stem cells (MSCs).^4–8^ The amniotic membrane (AM), an inner placental membrane that covers the developing fetus, comprises a single-cell epithelial layer residing on a basement membrane and an underlying stromal layer with sparse spindle-shaped fibroblasts and MSCs.^4^ A loose spongy layer between the two placental membranes allows for simple separation of amnion from chorion containing trophoblast (CM-TR). CM-TR is the outer membrane that encloses AM. It comprises a chorionic membrane (CM) structurally similar to that of AM and a trophoblast (TR) connected to the maternal decidua.^4^

The development of methods for isolation, processing, and preservation of placental membranes has led to the commercialization of AM and CM-TR. However, the majority of currently available placental products are derived from AM. Historically, AM has been frequently used for wound management and in surgical procedures due to ease of isolation and cleaning. At present, very few placental products containing CM are commercially available. Among them, only one cryopreserved CM retains all components of the fresh tissue, including viable cells. Clinical studies in chronic wound management have demonstrated that the use of either cryopreserved AM or CM containing viable cells results in similar wound closure rates.^9,10^ Although both membranes can be applied to wounds interchangeably, cryopreserved CM is soft and conforming, making it more suitable for filling tunneling or undermining wounds.^11^

Recently, a new lyopreservation method has been developed for AM that retains endogenous viable cells within the tissue.^12,13^ Importantly, this method allows for long-term storage of living cells and tissues at room temperature, overcoming the requirement for specialized ultralow temperature equipment for storage and transportation of cryopreserved cells and tissues. This requirement is the main limitation of cryopreservation methods. Previously reported methods of lyophilization did not allow for retention of viable cells in the tissue, making these methods nonapplicable to living cell and tissue preservation. A scientific study demonstrated that the structure, cell viability, and functional properties of lyopreserved AM are similar to those of both cryopreserved and fresh AM.^12^ Clinically, both lyopreserved and cryopreserved AMs containing viable cells have similar closure rates in the treatment of chronic wounds.^14^ These results suggest equivalency between cryopreserved and lyopreserved AMs with the added convenience of room-temperature storage and distribution of the lyopreserved AM.

In this study, we investigated the applicability of lyophilization for the preservation of CM with viable cells. The main objective of this study was to characterize the structural and functional properties of viable lyopreserved chorionic membrane (VLCM). A second objective was to investigate the immunogenicity of CM-TR, CM, and TR, an important question that has not been fully addressed.

## Clinical Problem Addressed

Long-term storage of living cells and tissues is heavily dependent on cryopreservation. In this study, we demonstrate the applicability of a recently developed lyophilization method for the preservation of all components in CM including viable cells. VLCM circumvents the requirement of “cold chain” storage and distribution, expanding its availability to wound care settings without capability to support “cold chain” logistics. Furthermore, addressing the immunogenicity of CM opens an avenue for wider acceptance of CM for clinical applications.

## Materials and Methods

### Tissue procurement, processing, and preservation

#### Placental tissue procurement

Placental tissue was collected from eligible donors and purchased from The National Disease Research Interchange (NDRI, Philadelphia, PA) and Cord Blood America, Inc. (CBA, Las Vegas, NV). NDRI and CBA provided the tissue procurement and ethics statements.^12^

#### Placental tissue processing

Placental tissues were aseptically processed as described previously.^12^ CM-TR, which is composed of the mesenchymal layer (CM) and the TR layer, was separated from placental AM, umbilical cord, and decidua by blunt dissection followed by a wash in anticoagulant citrate dextrose solution A (Fenwal, Inc., Lake Zurich, IL). CM-TR was then subjected to enzymatic treatment with dispase solution (Corning, Inc., Corning, NY) to allow for separation of CM from TR. CM was washed with saline and mechanically cleaned to remove residual blood and TR. CM was then incubated for 24–48 h in an antibiotic cocktail solution as described previously.^12^ CM was rinsed in Dulbecco's phosphate-buffered saline (DPBS) (Life Technologies, Carlsbad, CA) and cut into 25 cm^2^ pieces.

Fresh samples of whole placenta, CM-TR, TR, fresh AM and CM, and viable cryopreserved amniotic membrane (VCAM) and viable cryopreserved chorionic membrane (VCCM) derived from the same donor were used for immunogenicity experiments. Fresh CM, VCCM, and VLCM derived from the same donor were used for other experiments.

#### Cryopreservation and lyopreservation

VCAM and VCCM were cryopreserved in FP-90 bags (Charter Medical, Winston-Salem, NC) containing 5% dimethyl sulfoxide (DMSO) (Sigma-Aldrich, St. Louis, MO) and 1% human serum albumin (Octapharma, Hoboken, NJ) in saline solution. All samples were stored at −80°C before thaw and use for experiments.

VLCM was prepared by incubating CM in lyoprotectant solution containing 0.5 M trehalose dihydrate (Avantor, Center Valley, PA) in DPBS for a minimum of 30 min followed by a DPBS rinse. The tissue was then lyophilized in vials using a LyoStar II lyophilizer (SP Scientific, Gardiner, NY). Lyophilization was performed as previously described.^12^ In brief, samples were subjected to an initial freezing temperature of −50°C for 120 min, followed by primary drying at 200 mTorr and −20°C for 360 min. Secondary drying was accomplished in three steps: 0°C for 360 min, 20°C for 360 min, and 30°C for 2,760 min. Chamber pressure was held at 200 mTorr throughout the drying cycles.

#### Rehydration and thawing procedure

VLCM was rehydrated in 5–10 mL of DPBS. FP-90 bags containing VCAM and VCCM in the cryopreservation solution were thawed in a water bath at 37 C. The cryopreservation solution was removed, and the membranes were rinsed in DPBS before use in experiments.

### Immunogenicity testing

#### Mixed lymphocyte reaction

Immunogenicity of fresh samples of CM-TR, AM, and CM was tested in an *in vitro* mixed lymphocyte reaction (MLR) assay. For this assay, cells from placental tissues were isolated using a 280 U/mL collagenase type II solution (Worthington Biochemical Corporation, Lakewood, NJ). Tissues were treated for 60–90 min at 37°C. The resulting cell suspensions were filtered through a 100 μm filter to remove tissue debris and washed twice with DPBS. Prepared placental-derived cells were mixed with allogeneic human leukocyte antigen (HLA) unmatched human peripheral blood mononuclear cells (hPBMCs) (SeraCare Life Sciences, Milford, MA) at a 1:5 ratio (0.2 × 10^6^ placental cells: 1 × 10^6^ hPBMCs) in 24-well culture plates in Dulbecco's minimal essential medium (DMEM) (Thermo Fisher Scientific, Waltham, MA) supplemented with 5% fetal bovine serum (FBS) (Thermo Fisher Scientific). These samples were then incubated for 4 days at 5% CO_2_, 95% humidity, and 37°C. hPBMCs alone were used as a negative control and a mixture of hPBMCs derived from two HLA unmatched donors (1:1 ratio) was used as a positive two-way MLR control. After 4 days, cocultured cells were collected and lysed in lysis buffer (Sigma-Aldrich) supplemented with a protease inhibitor cocktail (Roche, Basel, Switzerland). Interleukin (IL)-2Rα was used to evaluate the activation of T cells in PBMCs in response to HLA and other immunogenic molecules expressed by allogeneic cells. IL-2Rα was measured in cell lysates using the sIL-2Rα ELISA kit (R&D Systems, Minneapolis, MN). A total of six donors were tested in the MLR experiments versus hPBMC derived from two donors.

#### Lipopolysaccharide challenge assay

Placental tissue sample pieces 4 cm^2^ (AM with CM-TR, TR, AM, and CM) were added to individual wells of a 24-well tissue culture plate containing 1 mL of DMEM (Thermo Fisher Scientific) and 5% FBS (Thermo Fisher Scientific). The samples were treated with 1 μg/mL bacterial lipopolysaccharide (LPS) (Sigma-Aldrich) for 20–24 h, collected, and assayed for the presence of tumor necrosis factor α (TNF)-α using a TNF-α ELISA kit (R&D Systems) following the manufacturer's protocol. hPBMCs (SeraCare Life Sciences) were used as a positive control in this assay, whereas hPBMCs and placental tissue samples without LPS were included as baseline TNF-α controls. A total of six donors were tested for the LPS challenge assay.

#### C57BL/6 mouse subcutaneous pocket implantation model

Ten-week-old C567BL/6 mice (Envigo Laboratories, Madison, WI) were housed at Noble Life Sciences vivarium in Sykesville, MD. The protocol and all procedures involving the care and use of animals were approved by the Noble Life Sciences' Institutional Animal Care and Use Committee. All procedures were performed in accordance with the guidelines and regulations of The Association for Assessment and Accreditation of Laboratory Animal Care International. All mice were fed a standard chow diet.

Two 0.5-cm skin excisions were created on the dorsum of each anesthetized animal (one excision on both sides of the midline). Placental tissue 1 cm^2^ was implanted into the subcutaneous pocket created postexcision. AM 1 cm^2^ served as a negative (nonimmunogenic) control. CM-TR 1 cm^2^ or CM 1 cm^2^ served as the test articles. Subcutaneous pockets not receiving any tissue material served as the sham control. Subcutaneous pockets were closed using skin glue. Animals were humanely euthanized 72 h postprocedure. Tissues at the implant site were harvested and fixed in formalin for further processing and histological evaluation. Tissue embedding, sectioning, and hematoxylin and eosin (H&E) staining were performed by Histoserv, Inc. (Germantown, MD). Tissue sections were evaluated microscopically, and images were captured at 4 × and 10 × .

### Evaluation of fresh CM, VLCM, and VCCM composition

#### Histological analysis

Two samples derived from three different donors for VLCM (postrehydration), VCCM (post-thaw), and fresh CM from three donors were fixed in 4% paraformaldehyde and processed for paraffin embedding. Standard protocols for tissue sectioning and staining were performed by Histoserv, Inc. Representative images of H&E- and Masson's trichrome (MT)-stained samples were captured using a photo camera (Canon, Inc., Ōta, Tokyo) mounted on an Olympus BH2 microscope (Olympus Co., Shinjuku, Tokyo). Histological scoring of animal's tissue sections was performed by a blinded pathologist at Noble Life Sciences.

#### Cell viability

Tissue staining for evaluation of cell viability was performed as previously described.^12^ Sixteen-millimeter diameter samples of fresh CM, VCCM, and VLCM from six donors were stained using a LIVE/DEAD Viability/Cytotoxicity Kit for mammalian cells (Thermo Fisher Scientific). Images were captured, and cells on captured images were counted using an EVOS FL Auto Imaging System using the Celleste Image Analysis software (Thermo Fisher Scientific). The percentage of viable cells per microscopic field was calculated by analyzing LIVE/DEAD-stained tissue section images using the equation: (number of viable cells/number of total cells) × 100%.

#### Growth factors and cytokines in chorionic membrane

For evaluation of growth factor and cytokine profile, fresh CM, VCCM, and VLCM tissue lysates were prepared from three donors. The lysates were prepared using a tissue protein extraction reagent (Thermo Fisher Scientific) supplemented with complete protease inhibitor (Roche) and solubilized using a GentleMACS Octo Dissociator (Miltenyi Biotec, Bergisch Gladbach, Germany) followed by centrifugation at 14,000 rpm (16,000 rcf, 18-place fixed angle rotor; Eppendorf Centrifuge 5415C) for 15 min. The supernatants were collected and analyzed using a multiplex growth factor panel kit (LXSAHM; R&D Systems) on a Bio-Plex MAGPIX plate reader (Bio-Rad, Hercules, CA). The test panel included IL-4, IL-10, angiogenin (ANG), angiopoietin (ANGPT)-1, ANGPT-2, hepatocyte growth factor (HGF), stromal cell-derived factor-1α (SDF-1α), platelet-derived growth factor-AA (PDGF-AA), placental growth factor (PIGF), vascular endothelial growth factor (VEGF)-A, VEGF-C and VEGF-D, matrix metalloproteinase (MMP)-1, MMP-2, and MMP-9, and tissue inhibitor of metallopeptidase (TIMP)1, TIMP2, TIMP3 and TIMP4.

#### Anti-inflammatory activity of fresh CM, VLCM, and VCCM *in vitro*

The anti-inflammatory activity of fresh CM, VLCM, and VCCM was tested by the inhibition of TNF-α secretion by LPS-activated THP-1 cell line (ATCC TIB-202; Human Acute Monocytic Leukemia, Manassas, VA). To assess anti-inflammatory activity, samples of conditioned medium derived from fresh CM, VLCM, and VCCM were collected after overnight incubation of 25 cm^2^ tissue samples in 5 mL of DMEM (Thermo Fisher Scientific) supplemented with 10% FBS (Thermo Fisher Scientific) at 37°C with 5% CO_2_ in a humidified atmosphere. CM samples from three different donors were tested. Unstimulated and LPS-stimulated THP-1 cells were cultured in DMEM with 10% FBS and served as negative and positive controls, respectively.

A total of 5 × 10^5^ THP-1 cells per well in a 24-well plate were treated with 1 μg/mL LPS (*Escherichia coli*; Sigma-Aldrich) in the presence of 1 mL of fresh CM-, or VCCM-, or VLCM-derived conditioned medium in triplicates. After an overnight incubation at 37°C and 5% CO_2_ in a humidified atmosphere, samples were centrifuged at 14,000 rpm (16,000 rcf, 18-place fixed angle rotor; Eppendorf Centrifuge 5415C) to pellet THP-1 cells. Collected culture medium was assayed for TNF-α using a human TNF-α ELISA kit (R&D Systems). TNF-α levels from each treatment group were quantified and compared with TNF-α levels in unstimulated and LPS-stimulated THP-1 cells.

#### Angiogenic activity of fresh CM, VLCM, and VCCM *in vitro*

A human umbilical vein endothelial cell (HUVEC) tube formation assay was used to evaluate angiogenic activity of fresh CM, VLCM, and VCCM-derived conditioned media as previously described.^6^

Conditioned media were obtained by incubating fresh CM, VLCM, and VCCM tissues derived from three different donors in endothelial cell growth basal medium-2 (EBM-2; Lonza, Walkersville, MD) with 2% FBS (Thermo Fisher Scientific) for 3 days at 37°C and 5% CO_2_ in a humidified atmosphere. Fivefold diluted CM-conditioned media samples with a final FBS concentration of 0.4% were used in the experiments. EBM-2 and endothelial cell growth medium (EGM-2) supplemented with 0.4% FBS were used as controls.

HUVECs (Lonza) at passages 4 or 5 were seeded at a concentration of 2 × 10^4^ cells per well in the Matrigel-coated 6.4 mm diameter flat-bottomed plate in the presence of conditioned media derived from fresh CM, VLCM, or VCCM. HUVECs seeded onto Matrigel (Corning, Inc.) in EBM-2 (Lonza) or EGM-2 BulletKit (Lonza) containing angiogenic growth factors served as negative and positive controls, respectively. HUVEC cultures were incubated for 5 h at 37°C and 5% CO_2_ in a humidified atmosphere. Images of the wells were captured using EVOS FL Auto Imaging System (Thermo Fisher Scientific). A total of three images of representative random fields from triplicate wells (nine images total) were taken at 4 × magnification. The number of loops formed by HUVECs per microscopic field was counted using WimTube tube formation assay analyzer (Wimasis Image Analysis; wimasis.com) as previously described.^15^

### Rabbit abdominal adhesion model

#### Animals

New Zealand White rabbits were housed at Noble Life Sciences. The experimental protocol was approved by and performed in accordance with the Institutional Animal Care and Use committee of Noble Life Sciences. Rabbits were fed the Harlan Teklad Global High Fiber Diet #2031 (Harlan, Madison, WI).

#### VLCM testing

The rabbit abdominal adhesion model was developed and described previously.^16^ In brief, a rabbit abdomen was subjected to a 10-cm midline laparotomy. The surface of the cecum placed adjacent to the sidewall was abraded with sterile gauze until capillary hemorrhage was observed. The surface of the cecum facing the bowel was also abraded in a similar manner. Next, a 9-cm^2^ region of the peritoneum and abdominal transverse muscle was removed from the right lateral abdominal wall. The rabbit had two surgical injury sites, onto one of which a 25-cm^2^ piece of VLCM was applied and sutured in place to cover the abdominal wall defect. The second surgical site had no barrier graft applied. The muscle wall was then sutured closed, followed by closure of the skin. Postoperative care was performed twice daily for 10 days. Animals were euthanized 28 days postsurgery and the tissues at two abdominal injury sites were evaluated macro- and microscopically for severity of postsurgical adhesion formation.

### Statistical analysis

Descriptive statistics included the mean, standard deviation, and percentages. Whisker plot was used for analysis and presentation of MLR immunogenicity data. Student's *t*-test was used to determine the significance of differences between groups. A *p*-value <0.05 was considered significant.

## Results

### CM does not trigger immune and inflammatory responses *in vitro* and *in vivo*

**Figure 1.**
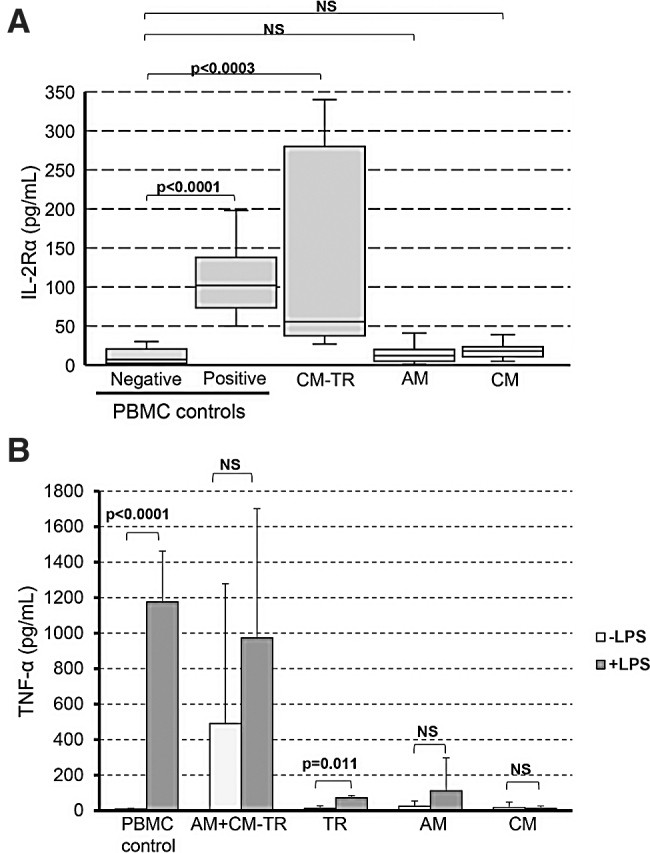
Evaluation of CM immunogenicity *in vitro*. **(A)** Cells from six different placental donors were isolated and tested in the MLR assay versus two PBMC donors. PBMCs were cultured in the presence of CM-TR, AM, or CM. PBMCs from a single donor cultured alone and a mixture of PBMCs from two donors served as negative and positive controls, respectively. Expression of IL-2Rα was used as a readout marker for immune cell activation in PBMC by the placental cells. The Whisker plot presents the MLR results. Placental cell immunogenicity was assessed by IL-2Rα expression increase versus PBMC alone (negative control). *n* = 6 donors in 12 MLR experiments. **(B)** Placental tissues derived from six donors were tested in the LPS challenge assay. This assay allows detection of TNF-α-secreting cells. LPS-induced TNF-α secretion served as a marker of immunogenic cells present in the tissues. LPS-treated PBMCs were used as a positive control in this assay, whereas PBMCs and placental tissue samples without LPS were included as baseline TNF-α controls. Bars show mean ± SD. The LPS-induced increase in TNF-α secretion was evaluated versus the baseline levels of TNF-α. AM, amnion; AM+CM-TR, amnion and chorion with trophoblast; CM, chorionic membrane without trophoblast; CM-TR, chorion containing trophoblast; IL, interleukin; LPS, lipopolysaccharide; MLR, mixed lymphocyte reaction; NS, not statistically significant; PBMC, peripheral blood mononuclear cells; SD, standard deviation; TNF-α, tumor necrosis factor-α; TR, trophoblast.

The immunogenicity of CM was investigated *in vitro* using two assays. For the assays, immunogenicity of CM was compared with immunogenicity of CM-TR, TR, and AM. The first assay was MLR. MLR is a standard assay for the evaluation of immune cell activation against allogeneic cells. Six placental donors were tested in MLR experiments. For MLR assay, cells were isolated from placental membranes (stimulators) and mixed with HLA-unmatched hPBMCs (responders). hPBMCs derived from two different donors were used in the MLR assay. hPBMC activation was detected by the expression of IL-2Rα, a marker of activated T lymphocytes, and one of the main cell subsets in hPBMCs.^17^ There was no statistically significant difference between the negative control (hPBMCs incubated alone) and hPBMCs cultured in the presence of cells isolated from fresh AM or CM (Fig. 1A). However, an immune response was detected in a mixture of two hPBMCs (positive control) and in a mixture of CM-TR cells with hPBMCs: as can be seen by the difference in IL-2Rα expression that was statistically significant (Fig. 1A).

TNF-α secretion by cells in placental tissue after stimulation with bacterial LPS was the second *in vitro* immunogenicity assay (also known as LPS challenge assay). This assay allows detection of TNF-α-secreting cells. TNF-α secretion is one of the characteristics of immunogenic cells.^18^ TNF-α levels in unstimulated hPBMCs (negative control), cleaned AM, CM, and isolated TR tissues were negligible. In contrast, AM+CM-TR tissue pieces had detectable levels of TNF-α (Fig. 1B). After stimulation with LPS, significant increase in TNF-α expression was detected in positive control (LPS-stimulated hPBMCs) and in the TR tissue samples. Increase in TNF-α was also detected in LPS-treated tissue samples comprising AM+CM-TR; however, this LPS-induced increase was not statistically significant as compared with the baseline levels of TNF-α in the AM+CM-TR samples (Fig. 1B). LPS did not trigger TNF-α secretion by the purified AM or CM (Fig. 1B).

**Figure 2.**
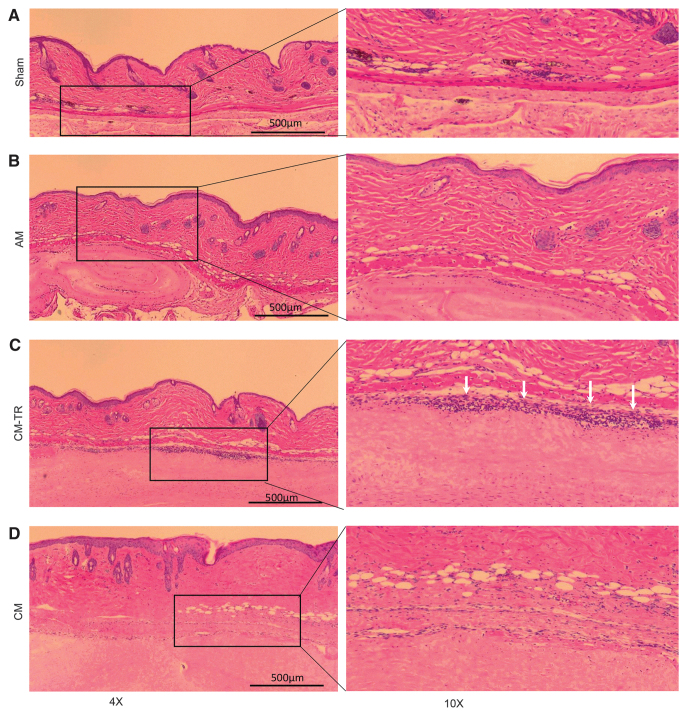
CM immunogenicity *in vivo*. Placental tissue immunogenicity was tested in the C57BL/6 mouse subcutaneous pocket implantation model. After 72 h postimplantation tissues from subcutaneous skin pockets were harvested, fixed, and processed for histological evaluation. H&E-stained tissue sections show small number of cells infiltrating the pocket area for the sham **(A)**, AM **(B)**, and CM **(D)**. In contrast, high number of cells infiltrating the interface between the mouse skin and placental tissue present in the pockets with implanted CM-TR (**C**, *white arrows*). Microphotographs at 4 × magnification are shown on the *left* (scale bar = 500 μm), and at 10 × magnification, *right panel*. H&E, hematoxylin and eosin.

*In vitro* results were confirmed *in vivo* using an implantation of placental tissues into subcutaneous pockets in C57BL/6 mice (Fig. 2). A magnitude of the inflammatory response was evaluated histologically by the number of cells infiltrating the area with implanted placental tissues compared with the sham control. Similar to the sham control (Fig. 2A), there were only a few cells infiltrating the area of the AM and CM implants (Fig. 2B, D), concluding that when implanted in subcutaneous pockets, AM or CM did not trigger an inflammatory response. On the contrary, the interface between the mouse tissue and the implanted CM-TR contained a substantial number of cells infiltrating the area (shown by white arrows) (Fig. 2C, right panel), suggesting that CM-TR triggers an inflammatory response.

In summary, results of *in vitro* and *in vivo* testing indicate that CM has low immunogenicity, whereas the TR layer may contain immunogenic cells that trigger inflammatory and immune responses.

### VCCM and VLCM retain tissue integrity of fresh CM

**Figure 3.**
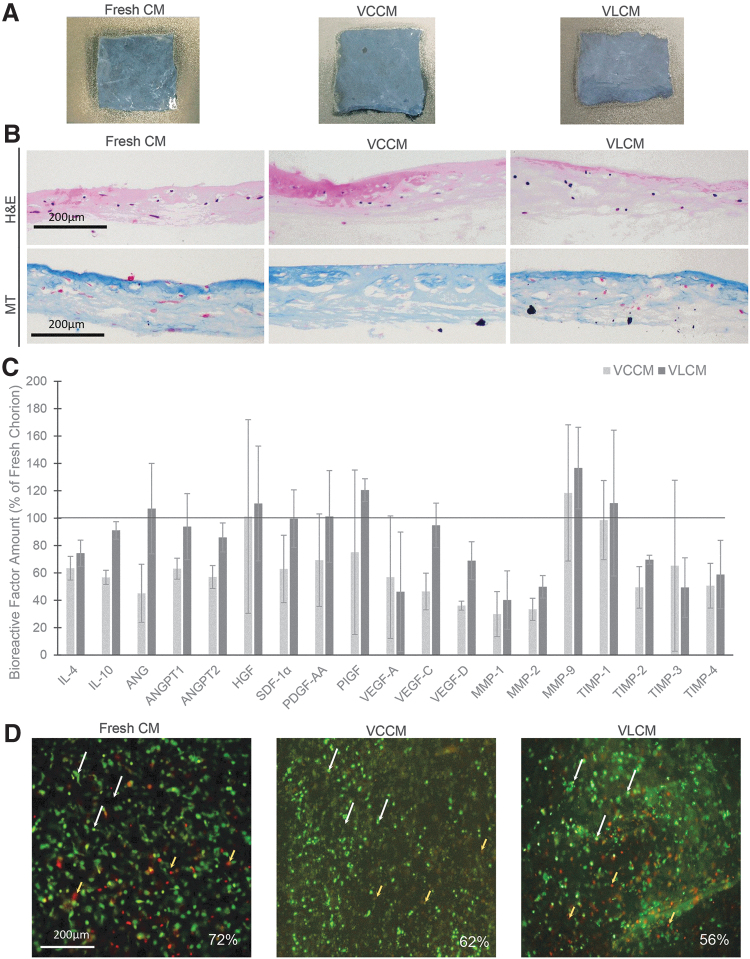
VCCM and VLCM retain integrity of fresh CM. **(A)** Visual appearance of fresh CM, VCCM, and VLCM. **(B)** Histological images of fresh CM (*left panels*), VCCM (*middle panels*), and VLCM (*right panels*): H&E (*top panels*) and MT (*bottom panels*) staining. Scale bar = 200 μm. **(C)** Levels of growth factors in VCCM and VLCM relative to fresh CM. Bars show mean ± SD growth factor levels for three donors. **(D)** Representative images of cell viability as assessed by LIVE/DEAD staining of fresh CM (*left panel*), VCCM (*middle panel*), and VLCM (*right panel*) derived from the same placental donor. Images show viable cells (*green*, *white arrows*) and dead cells (*reddish-yellow* and *red*, *yellow arrows*) in fresh CM, VCCM, and VLCM. Cell viability (%) is shown for each individual image (*n* = 3 donors). Scale bar = 200 μm. ANG, angiogenin; ANGPT, angiopoietin; HGF, hepatocyte growth factor; MMP, matrix metalloproteinase; MT, Masson's trichrome; PDGF-AA, platelet-derived growth factor-AA; PIGF, placental growth factor; SDF-1α, stromal cell-derived factor-1α; TIMP, tissue inhibitor of metallopeptidase; VEGF, vascular endothelial growth factor; VCCM, viable cryopreserved chorionic membrane; VLCM, viable lyopreserved chorionic membrane.

Fresh CM, after overnight antibiotic treatment, was cryopreserved or lyopreserved as described in the Materials and Methods section. Figure 3A shows similar visual appearance of fresh CM, VLCM postrehydration, and VCCM post-thaw. Blinded investigators were unable to distinguish VLCM from VCCM or fresh CM.

Histological staining with H&E and MT was used to evaluate the structural integrity of VLCM. The structure of VLCM was compared with that of fresh CM and VCCM tissues. Histologically, fresh CM, VCCM, and VLCM sections show a collagen-rich mesenchymal layer with sparse fibroblast-like cells across the tissue and absence of a TR layer, removed during tissue processing (Fig. 3B). H&E staining of thawed VCCM and rehydrated VLCM revealed that tissue architecture remained intact, as observed in fresh CM (Fig. 3B, top panels). MT staining demonstrated similar amount and distribution pattern of collagen throughout fresh CM, VCCM, and VLCM (Fig. 3B, bottom panels). These results suggest that neither preservation method altered tissue structure of native CM tissue.

Next, the presence of bioactive factors native to the stromal layer of fresh CM was confirmed in VCCM and VLCM. Both VCCM and VLCM retained endogenous CM factors that are known to play an important role in wound tissue repair (Fig. 3C). Comparing fresh CM, VCCM, and VLCM, there were no significant differences observed in the levels of IL-4 and IL-10; ANG; ANGPT-1 and ANGPT-2; HGF; SDF-1α; PDGF-AA; PIGF; VEGF-A, VEGF-C, and VEGF-D; MMP-1, MMP-2, and MMP-9; and TIMP1, TIMP2, TIMP3, and TIMP4 (Fig. 3C).

The presence of viable cells in fresh CM, VCCM, and VLCM was evaluated using the LIVE/DEAD cell viability assay. VCCM and VLCM samples were analyzed after several days, with the longest analyzed after 180 days storage at −80°C and room temperature, respectively. Tissue samples were stained with LIVE/DEAD fluorescent dyes to determine the presence of viable cells. The white arrows highlight the viable cells (green only). The yellow arrows illustrate the red and reddish-yellow dead cells. As shown in Fig. 3D, viable cells were present in fresh CM, VCCM, and VLCM. Overall, there were no significant differences in the percentage of viable cells between fresh CM, VCCM, and VLCM (Fig. 3D).

### Anti-inflammatory activity of fresh CM, VCCM, and VLCM *in vitro*

**Figure 4.**
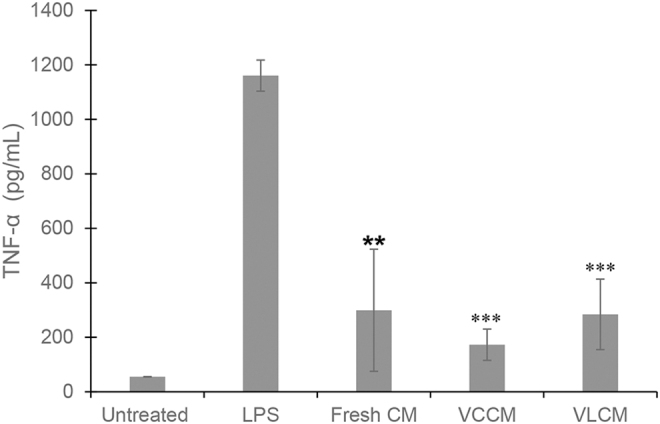
Anti-inflammatory activity of VCCM and VLCM *in vitro*. Anti-inflammatory activity of fresh CM, VCCM, and VLCM was evaluated by quantitating inhibition of TNF-α release from LPS-activated THP-1 cells. Unstimulated and LPS-activated THP-1 cells alone served as negative and positive controls, respectively. Student's *t*-test was used to determine differences between experimental groups with statistical significance. Bars show mean ± SD, **p < 0.01, ***p < 0.001 compared with the LPS-activated THP-1 cells. (*n* = 3 donors, three technical replicates).

Inhibition of TNF-α secretion by LPS-activated THP-1 cells in the presence of fresh CM-, VCCM-, and VLCM-derived conditioned media was used for evaluation of anti-inflammatory activity of fresh CM, VCCM, and VLCM. Exposure of LPS-activated THP-1 cells to conditioned media derived from fresh CM, VCCM, and VLCM resulted in a significant reduction in TNF-α levels as compared with LPS-activated THP-1 cells alone (positive control) (Fig. 4). These data suggest that (1) soluble factors secreted into culture medium derived from CM tissue samples inhibit TNF-α secretion and (2) VCCM and VLCM retain the inhibitory factors present in fresh CM.

### Angiogenic activity of VCCM and VLCM *in vitro*

**Figure 5.**
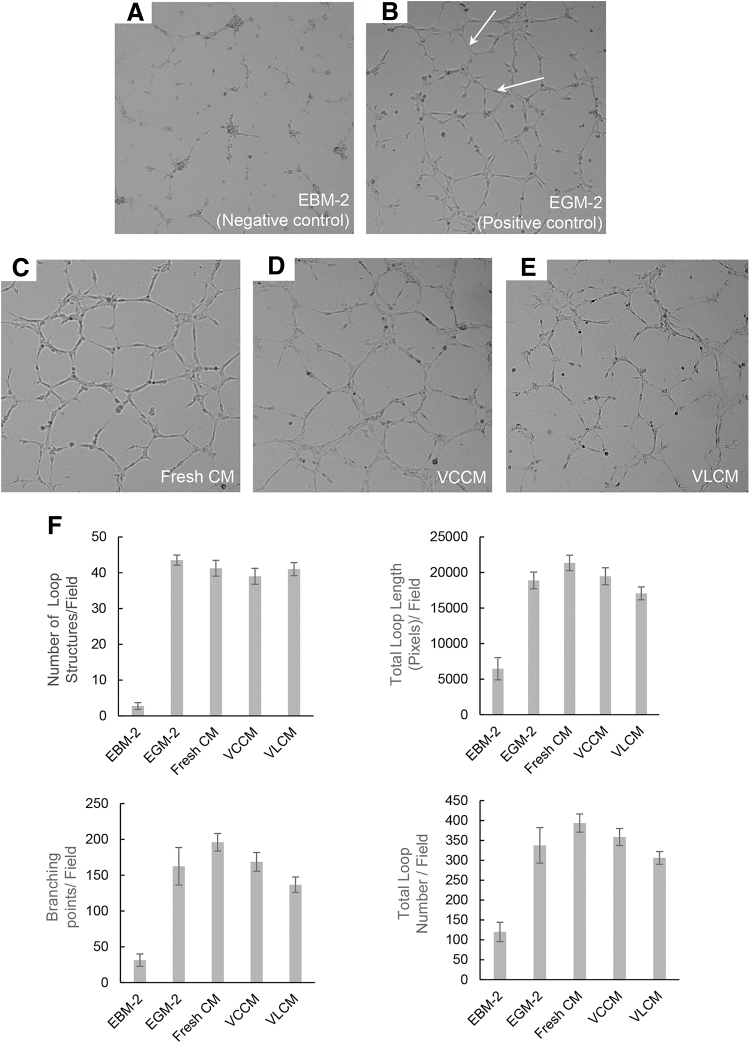
Proangiogenic activity of VCCM and VLCM *in vitro*. A HUVEC tube formation assay was used to evaluate proangiogenic activity of fresh CM-, VLCM-, and VCCM-derived conditioned media as described in the Materials and Methods section. Microphotographs show HUVEC loop structures formed in the presence of **(A)** EBM-2 or **(B)** EGM-2 media, or **(C)** Fresh CM-, **(D)** VCCM-, or **(E)** VLCM-derived conditioned media. **(F)** Quantitative analysis of HUVEC loop structures included the number of loop structures, total tube length, total branching points, and total number of tubes per microscopic field. (*n* = 3 donors). Bars show mean ± SD. EBM-2, endothelial cell growth basal medium; EGM-2, endothelial cell growth medium; HUVEC, human umbilical vein endothelial cell.

To determine the angiogenic activity of VCCM and VLCM *in vitro*, we used the HUVEC angiogenic tube assay. In brief, HUVECs seeded onto Matrigel were incubated for 5 h with fresh CM-, VCCM-, and VLCM-derived conditioned media. Culture plates with HUVEC were analyzed microscopically for the formation of closed loop structures, an indication of new blood vessel formation (Fig. 5). EBM-2 served as the negative control (Fig. 5A). Fresh CM, VCCM, and VLCM conditioned media promoted the formation of closed loops by HUVECs (Fig. 5C**–**E). These results are comparable with that observed for HUVECs cultured in EGM-2 (positive control) that has a mixture of angiogenic factors (Fig. 5B). Quantitative analysis demonstrated no significant differences in the number of loops, branching points, total length, and area of formed loop structures in fresh CM-, VCCM-, and VLCM-conditioned media (Fig. 5F). Taken together, these data show that condition media derived from fresh CM, VCCM, and VLCM contain proangiogenic growth factors.

### VLCM prevents postsurgical adhesion formation in a rabbit abdominal adhesion model

**Figure 6.**
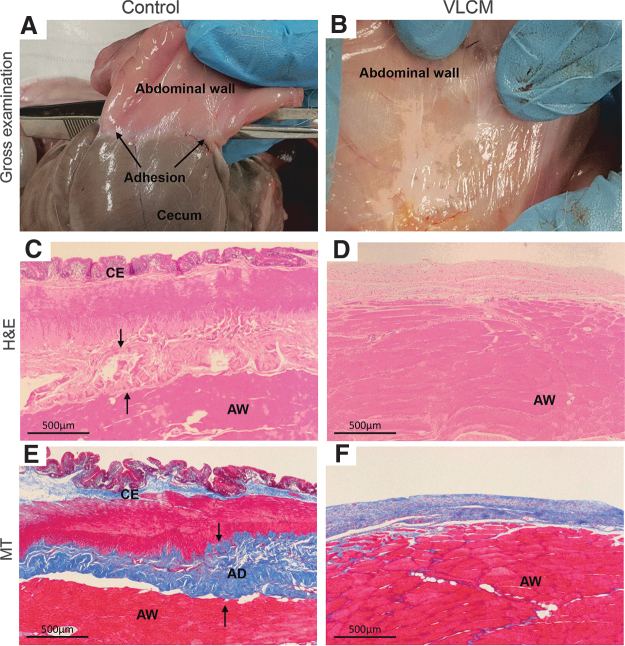
Evaluation of VLCM antifibrotic activity in a rabbit abdominal adhesion model. Tissues were collected at 28 days postsurgery and were evaluated macro- and microscopically for severity of postsurgical adhesion formation. Postsurgical gross evaluation of the abdominal area shows that **(A)** the cecum has tight adhesions to the AW at the control site (no graft, *black arrows* point to adhesions), **(B)** whereas adhesions were not detected at the site that received VLCM. Histological evaluation: **(C)** H&E-stained tissue sections of control and **(D)** VLCM-treated sites. *Black arrows* point to a dense collagen band formed at the control site. **(E)** The presence of fibrous tissue between the cecum and AW was seen in the MT-stained section of the control site. **(F)** There was no fibrous tissue between the cecum and AW detected at the VLCM site. AD, adhesion; AW, abdominal wall; C, cecum. (*n* = 4).

VLCM was tested in the rabbit abdominal adhesion model described previously.^16^ VLCM was applied to the injured area at one surgical site. A second surgical site in the same animal did not receive a graft and served as a control. Gross and histological evaluation of surgical sites was performed at day 28 postsurgery. Gross examination revealed adhesion formation between the cecum and abdominal wall at control sites (Fig. 6A), whereas there were no adhesions at VLCM-treated surgical sites (Fig. 6B). Grades of fibrosis between abdominal wall and cecum and inflammation were scored by a blinded third-party pathologist (Table 1). Histologically, control sites revealed the formation of a dense collagen band, representing an adhesion, between the cecum and abdominal wall (Fig. 6C, E and Table 1). The adhesion correlated with the inflammation: the surgical site was infiltrated with significant number of cells. Grade 1 minimal fibrosis on the injured abdominal wall was observed at VLCM-implanted sites. Weak adhesion with minimal inflammation (a few cells at one focus) was formed between the abdominal wall and VLCM, but not between the abdominal wall and the cecum (Fig. 6D, F and Table 1). By day 28, VLCM was not detectable (neither by gross nor by microscopic evaluation) at the implantation site (Fig. 6B).

**Table 1.** Peritoneal adhesion scoring

Parameters	Score at Day 28	
	Control	VLCM
Adhesion between wall and cecum (gross evaluation)	3	0
Fibrosis (histological evaluation)	3	1^a^
Inflammation (histological evaluation)	3	1^a^

Grading scale: 0, normal; 1, minimal; 2, mild; 3, moderate; 4, severe.

^a^ Abraded abdominal wall with no adhesion to cecum.

VLCM, viable lyopreserved chorionic membrane.

## Discussion

This study addressed the immunogenicity of CM-TR, a chorionic placental membrane comprising the mesenchymal (CM) and TR layers. Results of *in vitro* and *in vivo* immunogenicity testing performed in the study show that CM-TR elicits an immunogenic response, whereas purified CM alone did not (Fig. 1). This observation can be explained by TR cell immunogenicity: for example, formation of antibodies against TR antigens has been detected during pregnancy.^19^ In contrast, the proximity of maternal decidua to TR suggests that contamination of the chorion with maternal blood cells during membrane isolation is another probable reason for the observed immunologic response as previously reported by Bailo *et al.*^20^ Bailo *et al.* demonstrated that unfractionated chorionic cells do trigger an immune response in the MLR assay; however, after removal of blood cells using CD45 and glycophorin A antibodies, chorionic cells did not induce hPBMC activation in MLR.^20^ In contrast to TR, CM and AM neither trigger hPBMC activation in the MLR assay nor secrete TNF-α after LPS challenge (Fig. 1). Results of the *in vivo* implantation of CM and CM-TR in subcutaneous pockets in mice are in line with *in vitro* experimental results. CM-TR triggered an inflammatory response that was detected by high number of cells infiltrating the interface between mouse tissue and the placental graft. In contrast, CM did not trigger an inflammatory response (Fig. 2). Currently, cryopreserved CM is being clinically used for management of wounds and in surgeries. There are no CM-related adverse events reported up to date.^4,11,21,22^

The main objective of this study was to evaluate VLCM, a lyopreserved TR-free chorion stored at room temperature, as an alternative to cryopreserved CM. Our results demonstrate that both VLCM and VCCM retain structural integrity of native tissue, including extracellular matrix, growth factors, and endogenous viable cells (Fig. 3). Histologically, tissue sections prepared from fresh CM, VCCM, and VLCM show a similar tissue architecture of the mesodermal layer and confirm the absence of TR, removed during tissue processing (Fig. 3B). Placental tissue is a rich source of anti-inflammatory cytokines, growth factors, and inhibitors of proteases.^1,23–26^ The levels of various bioactive factors in both VCCM and VLCM were comparable with those in fresh CM (Fig. 3C), demonstrating that neither cryopreservation nor lyopreservation of fresh CM destroys the native components of fresh tissue (Fig. 3C). Evaluation of live and dead cells in the tissues using fluorescence staining confirmed the presence of viable cells in all samples without significant differences between fresh CM, VCCM, and VLCM (Fig. 3D). Recent literature has shown the added benefit of preserving endogenous viable cells together with native tissue matrix and bioactive factors.^27^

*In vitro* experiments confirmed that anti-inflammatory and angiogenic properties of fresh CM are retained in both VCCM and VCLM (Figs. 4 and 5). Conditioned media derived from fresh CM, VCCM, or VLCM showed a significant reduction in TNF-α secretion by LPS-activated monocytic THP-1 cells (Fig. 4). As high levels of TNF-α correlate with nonhealing of chronic wounds, a decrease in TNF-α is a positive predictive factor for wound closure.^28,29^ In our *in vitro* angiogenesis assay, we used four parameters to evaluate different aspects of loop formation by HUVECs: average length, number, area, and number of branch points. We found that all four parameters were higher upon exposure to conditioned media derived from fresh CM, VCCM, or VLCM than those of the negative control. The magnitude of this effect was not different between fresh CM-, VCCM-, and VLCM-derived condition media and with EGM-2, the positive control containing angiogenic growth factors (Fig. 5). Several growth factors are required in loop formation by HUVECs, such as VEGF, basic fibroblast growth factor, insulin-like growth factor, and epidermal growth factor.^30,31^ The observation that CM, VCCM, and VLCM have a positive effect on all four parameters of loop formation demonstrates that the required cocktail of growth factors to promote angiogenesis is present and functional in conditioned media. Although the anti-inflammatory and angiogenic properties of AM have been investigated previously, similar studies on chorion have not been reported. The present data are the first to show that CM has anti-inflammatory and angiogenic properties.

Fresh and cryopreserved AM have been shown to reduce fibrosis.^26,32,33^ Reduction in tissue inflammation and rapid tissue healing are imperative for adhesion prevention.^34,35^ AM has these properties that might mediate AM antifibrotic effects. Using a variety of animal models, both reduction of scarring and prevention of postsurgical adhesions have been reported for AM.^36,37^ However, several studies demonstrated that dehydration of AM resulted in alteration of tissue matrix and growth factors, and led to a significant decrease of AM antifibrotic activity.^38,39^ Our *in vitro* results showed that VLCM is both anti-inflammatory and proangiogenic. Therefore, our next step was to investigate whether VLCM was effective for antifibrotic activity. Results of VLCM testing in a rabbit abdominal adhesion model demonstrated that VLCM implanted at the surgical site prevented formation of postsurgical adhesions (Fig. 6). These data obtained in a clinically relevant animal model suggest the potential utility of VLCM as a bioactive barrier with anti-inflammatory, proangiogenic, and antifibrotic properties.

In summary, our study demonstrates that isolated TR-free CM is nonimmunogenic. We found that similar to VCCM, VLCM retains native components and properties of fresh CM tissue. Taken together, these data demonstrate the applicability of our lyopreservation method for preservation of all components of the chorion, including endogenous viable cells with the added benefit of long-term room temperature storage.

## Innovation

This study demonstrates low immunogenicity of CM supporting its clinical use. For the first time, the structural and functional properties of fresh CM, VCCM, and VLCM were investigated. Results demonstrate that CM can be cryopreserved or lyopreserved without alterations of native tissue components, including viable cells and functional properties. Data support structural and functional equivalency of VLCM and VCCM.

**Key Findings**

- TR-free human chorionic membrane is nonimmunogenic.
- The described lyopreservation method is applicable for preservation of all components of chorionic membrane: it maintains the tissue architecture and retains endogenous viable cells and growth factors of fresh chorionic tissue.
- Structural and functional assays demonstrate that both VCCM and VLCM retain inherent components and properties of fresh CM supporting equivalency of both preservation methods.
- VLCM prevents postsurgical adhesion formation in a rabbit abdominal adhesion model.

## About the Authors

**Malathi Sathyamoorthy, PhD,** is currently the Director of Research and Development at OTI. She earned her PhD in Biochemistry and Molecular Biology from University of Maryland, Baltimore, MD. **Sandeep Dhall, PhD,** is currently a senior scientist at Osiris Therapeutics, Inc. (OTI). He obtained his PhD in Bioengineering and postdoctoral training in Cell Biology and Neuroscience from University of California, Riverside, CA. **Nicholas Johnson, BS,** is a senior research associate at OTI. **Vimal Jacob, BS,** is a research associate at OTI. **Anne Lerch, BS,** is a senior research associate at OTI. **Brielle Jones, MS,** is a scientist at OTI. **Alla Danilkovitch, PhD,** is chief scientific officer at OTI. She earned her PhD in cell biology from Moscow State University, Russia. She has >25 years of research experience in stem cells, immunology, and cancer biology.

## Abbreviations and Acronyms

AM: amniotic membrane
ANG: angiogenin
ANGPT: angiopoietin
bFGF: basic fibroblast growth factor
CM: chorionic membrane without trophoblast
CM-TR: chorion containing trophoblast
DMEM: Dulbecco's minimal essential medium
DPBS: Dulbecco's phosphate buffered saline
EBM-2: endothelial cell growth basal medium
EGM-2: endothelial cell growth medium
FBS: fetal bovine serum
H&E: hematoxylin and eosin
HGF: hepatocyte growth factor
HLA: human leukocyte antigen
hPBMC: human peripheral blood mononuclear cell
HUVEC: human umbilical vein endothelial cell
IGF: insulin-like growth factor
IL: interleukin
LPS: lipopolysaccharide
MLR: mixed lymphocyte reaction
MMP: matrix metalloproteinase
MSC: mesenchymal stem cell
MT: Masson's trichrome
mTorr: millitorr
NS: not statistically significant
PDGF-AA: platelet-derived growth factor-AA
PIGF: placental growth factor
SD: standard deviation
SDF-1α: stromal cell-derived factor-1α
TIMP: tissue inhibitor of metallopeptidase
TNF-α: tumor necrosis factor α
TR: trophoblast
VCAM: viable cryopreserved amniotic membrane
VCCM: viable cryopreserved chorionic membrane
VEGF: vascular endothelial growth factor
VLCM: viable lyopreserved chorionic membrane
